# Transformer-Convolution Pyramid Network for Multi-Class Kidney, Tumor, and Cyst Segmentation

**DOI:** 10.3390/cancers18203287

**Published:** 2026-10-12

**Authors:** Evgin Goceri

**Affiliations:** Biomedical Engineering Department, Akdeniz University, 07070 Antalya, Türkiye; evgin@akdeniz.edu.tr

**Keywords:** CNN, cyst segmentation, deep network, kidney segmentation, transformer, tumor segmentation

## Abstract

**Background/Objectives**: Chronic kidney diseases are fatal disorders, and early treatment is crucial for reducing mortality. Segmenting kidneys, tumors, and cysts from computed tomography scans has a significant role in assessing these diseases’ progression, determining their severity, and planning surgeries. However, accurate segmentation is challenging for many reasons. The objective of this study is to develop a hybrid network that can overcome these challenges. **Methods**: The proposed network was designed using (i) a patch-joining and swin transformer block to capture global features efficiently, (ii) a Dynamic Channel Attention block to emphasize significant feature locations with the help of channel-wise attention, (iii) a block to perform complementary fusion of features coming from different layers, (iv) an edge-detection block to improve the performance by paying extra attention to edge features, and (v) an efficient activation function to overcome dying neuron and bias-shifting problems caused by the commonly used rectified linear unit. Extensive experiments were conducted using images from KiTS-2023 and Kaggle databases. Also, state-of-the-art techniques were applied to identical datasets and evaluated using identical metrics for fair comparisons. In addition to the commonly used Dice Similarity Coefficient (DSC), the Normalized Surface Distance (NSD) was used for evaluation. **Results**: The proposed method yields DSC/NSD scores of 98.35 ± 0.65%/97.62 ± 0.55%, 94.88 ± 0.57%/93.84 ± 0.50%, and 81.99 ± 0.45%/80.93 ± 0.40% for kidneys, tumors, and cysts, respectively. **Conclusions**: The results indicate the superiority of the proposed approach in segmenting kidneys, tumors, and cysts. The proposed method has the potential to support medical experts by providing quantitative and objective segmentation results.

## Simple Summary

Segmenting kidneys, tumors, and cysts from computed tomography scans is essential for assessing chronic kidney disease progression and severity and for surgical planning. However, their accurate segmentation is challenging for many reasons, including unclear edges, noise, artifacts, and inhomogeneous intensities in the images. To address these challenges, a hybrid network combining the advantages of convolutional operations and transformers was developed. The proposed method achieves higher segmentation performance for kidneys, tumors, and cysts than state-of-the-art methods. It can assist medical experts in performing rapid and reliable assessments, evaluating disease severity and progression, and planning surgeries and treatments based on quantitative and objective results. By reducing subjectivity and the limitations of conventional methods that rely on visual examination of images, it can improve the reliability of these assessments. Additionally, it can reduce the workload of medical experts, allowing them to devote more time to other clinical tasks.

## 1. Introduction

This section introduces the research problem and the objectives of the study (Section 1.1), discusses the motivation behind the proposed work (Section 1.2), and explains the novel contributions of this study (Section 1.3).

### 1.1. Background

Tumors or cysts that develop in the kidneys can severely impair kidney function and are serious diseases. Also, chronic kidney diseases are known as one of the most prevalent fatal disorders worldwide. It is estimated that, by 2040, they will become the 5th leading cause of death, with 5 to 10 million deaths per year [1,2]. These diseases may be caused by many factors, such as hypertension, diabetes, ethnicity, gender, age, and smoking [1,2]. Early detection and treatment are crucial in reducing mortality from these diseases. In assessing their progression and severity, as well as in surgery and treatment planning, segmentation of the kidney, its tumor, and cyst from computed tomography (CT) images plays a significant role [1,2]. However, precise segmentation of kidneys based on visual examinations of the images is difficult for many reasons (e.g., noise, artifacts, and inhomogeneous intensities in the images). Also, conventional segmentation needs expertise, knowledge and experience. In addition, it is time-consuming, not reproducible, and a laborious task. Furthermore, the manual segmentation approach is subjective and can lead to intra-/inter variabilities, inaccurate, and late diagnosis. Moreover, kidney cysts and tumors have unclear edges, various morphologies, and unpredictable locations. These factors make their segmentations much more difficult [1,3]. Therefore, computer-assisted automated methods are needed for precise, accurate, objective, and fast segmentations based on quantitative evaluations of the images.

### 1.2. Motivation

In the literature, mostly Convolutional Neural Networks (CNNs) have been implemented (Section 2) due to their efficiency in the detection and extraction of details by using various pooling and convolution layers. However, they are not adequate and have limitations in capturing and modeling the long-range global contextual information. This is because they disregard global features by focusing solely on a local set of pixels (i.e., pixel-wise information) because of the intrinsically local nature of convolution operations [4]. Recently, transformer-based networks, including a self-attention mechanism, have been proposed to obtain long-range semantic dependencies and solve the issues caused by CNNs (Section 2). Transformers do not need convolutional layers, unlike CNNs; they use a dispense-convolution operator and rely only on self-attention mechanisms for modeling the global contextual feature information. Even though transformers are good at extracting global information with the help of global receptive fields, they lack interaction with neighboring features due to partitioning the image into fixed-size patches and processing them separately while modeling local features [5]. Because of this, they ignore semantic relations among regions, are not successful in extracting and modeling local characteristic information (local details), and lead to poor performance. Therefore, methods with pure CNNs and transformers are not efficient.

Although hybrid architectures have been used to obtain both rich local details and global information, they have drawbacks or limitations in segmentating kidneys, tumors, and cysts (Section 2). Also, the Rectified Linear Unit (ReLU) is almost a default activation function in the literature, despite its tendency to cause dying neurons and bias effects. Additionally, models have been trained and tested on different datasets with different counts and variations in images, making it unclear which technique is the most effective. Furthermore, various metrics (e.g., Intersection over Union (IoU), Normalized Surface Distance (NSD) or Dice Similarity Coefficient (DSC)) have been used for evaluation. Therefore, comparing techniques based on different metrics is not meaningful. Moreover, most models in the literature focus solely on kidney, cyst, or tumor segmentation, leaving their effectiveness in segmenting all three unexamined. Only a few studies have aimed to develop a model capable of segmenting all three (Section 2). Because of the reasons mentioned above, there is a motivation for developing an effective technique for kidney, cyst and tumor segmentation from CT scans and to conduct an extensive and fair comparison of state-of-the-art approaches to determine the best technique.

### 1.3. Novel Contributions

In this work, inspired by recent studies, a new fully automated approach based on CNNs and transformers has been developed. In the proposed method, local details and global characteristic features in the images have been captured efficiently by utilizing the advantages of both vision transformers and deep convolutional networks. The proposed architecture has a pyramid shape and includes an encoder and decoder module. These modules have been constructed with residual convolutional blocks. Skip connections have been used in the proposed architecture because of their effectiveness in eliminating the vanishing gradient issue, advancing the performance of deep learning, and enhancing the generalization ability of the network [6,7]. Also, Dynamic Channel Attention (DCA) blocks have been designed and used in the architecture to emphasize significant feature locations with the help of channel-wise attention. In addition, the blocks named Complementary Fusion of the Features (CFF) have been designed and used to enhance segmentation performance by complementing those features in the stage of feature joining. Features obtained from different layers during the encoding and decoding have been provided by the DCA and CFF blocks. Furthermore, two Edge Detection (ED) blocks have been integrated to pay extra attention to the edge features within the images. In addition, a new block, namely Patch-Joining and Swin Transformer (PJST), has been designed and used in the encoding phase to capture global features efficiently. It utilizes the advantages of both swin transformers [8] and skip connections. It is of great significance to use swin transformers in this block due to their two properties: (1) They have the advantage of CNNs in capturing local details with local attention mechanisms. (2) They have the advantage of transformers in modeling long-range dependencies by using shifted window schemes. The shifted windows are alternately used with normal windows to keep the link among them and to provide information interaction (communication) among those windows. However, the alternating process leads to limitations in the application of local window-based attention and reduces the global modeling ability. Therefore, the PJST block has been developed to improve the information communication and global modeling ability of the proposed architecture.

The performance of the proposed approach has been investigated in segmenting kidneys, cysts, and tumors from CT scans. Additionally, to ensure a more reliable and consistent comparison, state-of-the-art methods in the literature have been applied to the same datasets, and their effectiveness has been assessed using identical evaluation metrics as the proposed method. This common evaluation protocol minimizes the influence of dataset-related variations and differences in metric selection, thereby enabling a more consistent assessment of the segmentation performance of the compared methods. In addition, evaluating these methods under a common experimental setting allows their performance in segmenting the kidneys, tumors, and cysts to be examined in a consistent manner. Thus, the comparative results provide a more reliable basis for assessing the relative performance of the proposed method. Briefly, the main contributions of this work are in six aspects:

1. A PJST module is introduced to address the limited global contextual modeling and insufficient interaction between neighboring image regions. The PJST module enables effective interaction among patch-level representations while exploiting the hierarchical self-attention mechanism of the swin transformer to capture both local and long-range contextual information. This facilitates the representation of complex anatomical structures and improves the discrimination of target regions from surrounding tissues.
2. DCA module is proposed to address the difficulty of identifying the most informative feature channels in the presence of heterogeneous and complex image characteristics. By dynamically modeling channel-wise dependencies and emphasizing discriminative feature responses, the DCA module strengthens relevant semantic representations while suppressing less informative features. This improves the network’s ability to distinguish pathological regions from visually similar surrounding tissues.
3. CFF module is developed to overcome the loss and inadequate integration of complementary information across different feature scales. The CFF module effectively combines complementary multi-scale features from different levels of the network, enabling the simultaneous utilization of detailed spatial information and high-level semantic representations. This enhances the representation of targets with varying sizes, shapes, and appearances and consequently improves segmentation accuracy.
4. ED modules are incorporated to address the difficulty of accurately delineating ambiguous and irregular object boundaries. By explicitly extracting and incorporating edge-related information into the segmentation process, the ED modules enhance boundary awareness and provide additional spatial constraints for the prediction of object contours. This contributes to more precise segmentation of regions with weak, indistinct, or irregular boundaries.
5. The proposed architecture integrates the PJST, DCA, CFF, and ED modules into a unified CNN–transformer-based segmentation framework, allowing their complementary capabilities to be exploited jointly. The effectiveness of the proposed design is comprehensively validated through quantitative experiments and comparisons with state-of-the-art methods, demonstrating its capability to achieve accurate segmentation of the target structures.
6. Meaningful comparisons and comprehensive results are presented. Recent techniques were employed on identical datasets and evaluated using the same metrics, thereby ensuring fair comparisons. Experimental results show that the proposed approach outperforms state-of-the-art methods in segmenting kidneys (98.35 ± 0.65%, 97.62 ± 0.55), tumors (94.88 ± 0.57%, 93.84 ± 0.50), and cysts (81.99 ± 0.45%, 80.93 ± 0.40) in terms of average DSC and NSD, respectively.

The remaining sections have been structured as follows: In Section 2, state-of-the-art methods in the literature are presented. In Section 3, the datasets used in our experimental work and the proposed hybrid method are outlined. In Section 4, the results are given. Section 5 presents the discussion, while Section 6 provides the conclusions of the study.

## 2. Related Work

Recently published review-type works indicated that mostly CNN-based models were used for segmentation of kidneys, tumors, and cysts from CT images [9,10,11,12,13,14]. In this work, except for the methods covered in [9,10,11,12,13,14], more recent methods have been examined, and significant information about them is presented in Table 1. In the literature, different architectures were designed, and different optimizers and loss functions were utilized. Although various activation functions were used, the most commonly used activation function in them was the ReLU. In addition, their performance was assessed in terms of different criteria (IoU or DSC).

The methods with pure CNNs [15,16,17] primarily rely on local convolutional operations, which limit their ability to model long-range contextual dependencies. Therefore, they are not efficient in obtaining both rich local details and global information, which are essential for accurate and precise segmentation of kidneys. The U-Net architecture, as a widely used CNN model, employs a symmetric encoder–decoder structure with skip connections that facilitate the transfer of spatial information from the encoder to the decoder, enabling precise localization of anatomical structures. Despite its effectiveness in extracting details, it is still heavily based on local convolutional operations; thus, it is ineffective in capturing global features.

To overcome these limitations, transformers have been integrated into CNNs. For instance, TransUNet combines CNN-based feature extraction with transformer-based global context modeling within a U-shaped encoder–decoder architecture [23]. By integrating convolutional features with transformer representations, TransUNet aims to capture both fine-grained spatial information and long-range dependencies. Similarly, UNETR employs a Transformer encoder to extract global representations from volumetric medical images and connects these representations to a convolutional decoder through multi-scale skip connections [24]. This design enables the integration of global contextual information with spatial features at different resolutions. However, transformer-based approaches may introduce considerable computational requirements, while the preservation of fine-grained spatial information remains an important consideration, particularly for small structures and lesions with indistinct boundaries.

The Swin-UNETR in [18] employs a hierarchical swin transformer encoder with shifted-window self-attention to learn multi-scale representations while maintaining a more localized attention mechanism than conventional global self-attention. Its U-shaped architecture incorporates multiresolution features into the decoding process, facilitating the integration of global contextual representations and spatially detailed information. Although it has demonstrated considerable potential in medical image analysis, its performance may still depend on the availability and diversity of training data, computational resources, and the ability to preserve subtle boundary information in challenging segmentation scenarios.

Architectures integrating CNNs and transformers at different stages of the encoder–decoder pipeline seek to improve feature representation across multiple spatial scales [18,19,20,21,22]. Nevertheless, effectively combining local and global representations while preserving spatial details throughout the decoding process remains an important architectural challenge. Therefore, they also have several drawbacks or limitations, such as poor global modeling ability caused by the swin transformer [18,19] suffering from over-segmentation [17,22]. Additionally, in [15,17,19,21], activations were provided by ReLU, which may lead to dying neurons and bias-shifting effects [25,26]. Therefore, it may cause poor performance when it is tested using datasets with more diversity of images.

These considerations are particularly relevant to kidney, tumor, and cyst segmentation in CT images, where substantial variations in size, morphology, intensity, and anatomical location can complicate the accurate delineation of target structures. While large anatomical structures may provide relatively extensive contextual information, small lesions, low-contrast cysts, and indistinct boundaries require effective preservation of fine-grained spatial features. Consequently, an effective segmentation framework should not only capture global contextual dependencies but also maintain detailed local representations and facilitate their efficient integration across multiple resolutions.

The above observations indicate that an efficient approach is still needed to achieve automated segmentation of kidneys, tumors, and cysts from CT scans. For this reason, in this work, a novel combined architecture has been developed. In the proposed architecture, activations have been performed using Leaky ReLU (L-ReLU). The proposed method adopts a hybrid CNN–transformer encoder–decoder framework to leverage complementary local and global feature representations. In contrast to relying exclusively on convolutional feature extraction or transformer-based global modeling, the proposed architecture incorporates three new blocks (DCA, CFF, and PJST) to facilitate multi-scale feature extraction, feature interaction, and information transfer between the encoder and decoder. This design is intended to improve the delineation of kidneys, tumors, and cysts exhibiting diverse morphological characteristics while addressing the challenges associated with small structures and ambiguous boundaries. The proposed framework is therefore positioned within the broader development of hybrid medical image segmentation architectures, with particular emphasis on the integration of complementary representations for multi-class kidney CT segmentation.

Moreover, the methods in the literature have been trained and tested with various datasets. In addition, different evaluation metrics have been employed to evaluate their performance. Therefore, comparisons according to the results presented in the publications are not fair. Because of this, in this work, the state-of-the-art techniques have been implemented, trained, and tested using the same datasets, and their performances have been evaluated based on the same metrics.

## 3. Materials and Methods

This section describes the datasets used in the experiments (Section 3.1) and presents the proposed hybrid method (Section 3.2).

### 3.1. Materials

The images used in our studies were taken from the publicly available KiTS-2023 database [27], which was constructed using data from the KiTS-2019 and KiTS-2021 databases. It contains a total of 590 CT images, comprising 110 test images and 480 training images. Five-fold cross-validation was employed in all experiments involving these images. The dataset was divided into five mutually exclusive folds at the patient level to prevent data leakage, ensuring that images (slices) from the same patient were not distributed across different folds. In each cross-validation iteration, four folds (480 images in total) were used for model development, while the remaining fold (110 images) was reserved for independent testing. Specifically, 20% of the images in the four-fold development set (96 images) were randomly selected for validation, and the remaining 80% (384 images) were used for training. Thus, each iteration consisted of 384 training images, 96 validation images, and 110 test images. This procedure was repeated five times, with each fold serving as the test set once. The final performance was reported as the mean and standard deviation across the five test folds.

To further investigate the effectiveness of the proposed method, an additional database publicly available on the Kaggle website [28] was incorporated into the study. Before dataset partitioning, all images underwent a quality-control screening process. Samples were excluded if they exhibited severe image artifacts, substantial image degradation, or missing or unusable segmentation annotations, as these issues could adversely affect the training and evaluation of the proposed model. After noisy samples were removed, a total of 890 CT images were retained, consisting of a test set of 210 images and a training set of 680 images. As with the KiTS-2023 dataset, five-fold cross-validation has also been applied in the experiments conducted, and the dataset was divided into five mutually exclusive folds at the patient level to prevent data leakage, ensuring that images (slices) from the same patient were not distributed across different folds. In each cross-validation iteration, four folds (680 images in total) were used for model development, while the remaining fold (210 images) was reserved for independent testing. Specifically, 20% of the images in the four-fold development set (136 images) were randomly selected for validation, and the remaining 80% (544 images) were used for training. Thus, each iteration consisted of 544 training images, 136 validation images, and 210 test images. This procedure was repeated five times, with each fold serving as the test set once. The final performance was reported as the mean and standard deviation across the five test folds. The split procedure is presented in Table 2.

### 3.2. Methods

**Figure 1:**
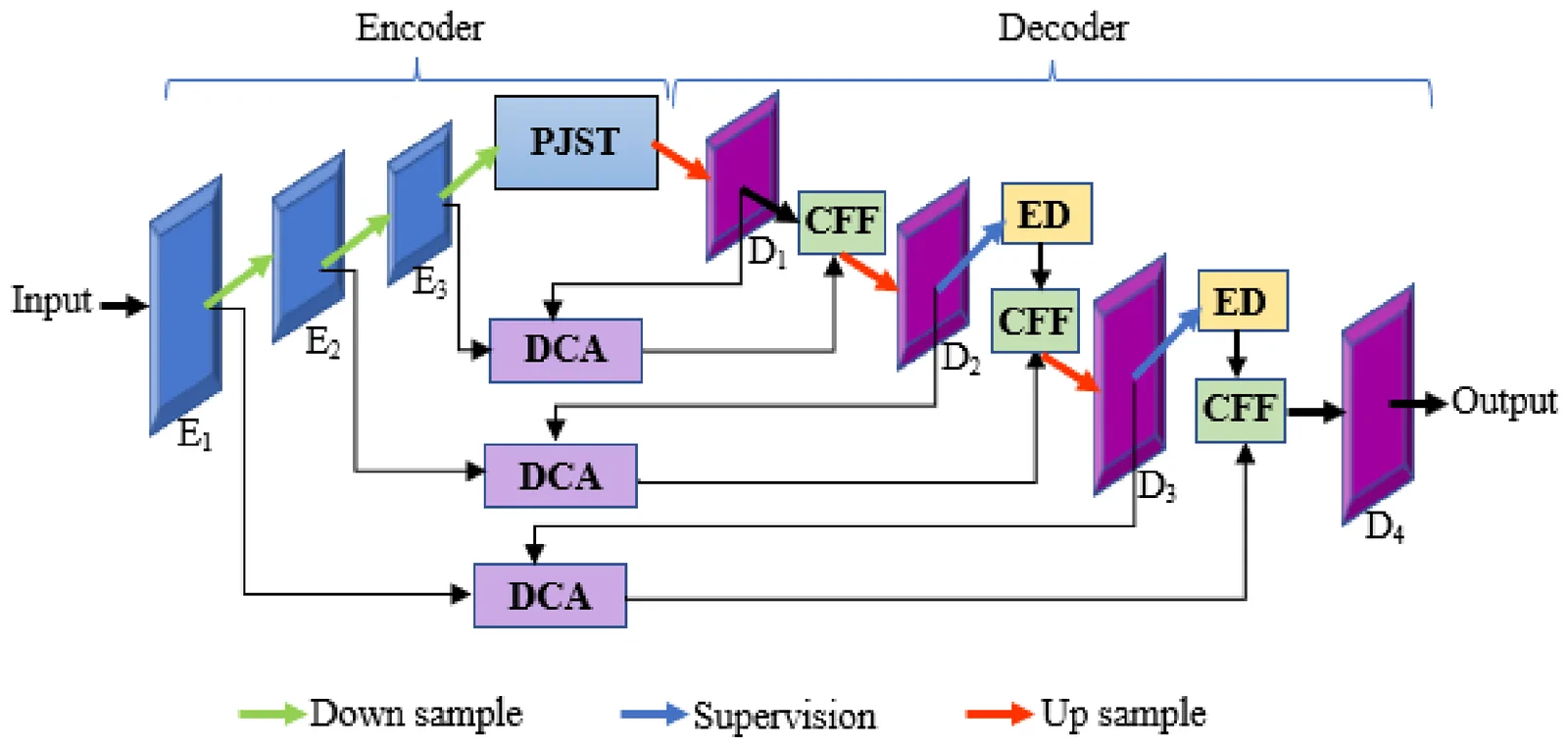
The proposed architecture.

In the proposed method, local and global features in the images have been efficiently captured by utilizing the advantages of both vision transformers and deep convolutional networks. The proposed architecture (Figure 1) has a pyramid shape and includes an encoder and decoder module (Section 3.2.1), which have been constructed with residual convolution blocks. Skip connections have been used in this network because of their effectiveness in eliminating the vanishing gradient issue, advancing the performance of deep learning, and improving the generalization ability of the network [6,7]. Also, DCA blocks (Section 3.2.2) have been designed and used in the network to emphasize significant feature locations with the help of channel-wise attention. In addition, CFF blocks (Section 3.2.3) have been designed and used to enhance segmentation performance by complementing those features in the stage of feature joining. The utilization of features obtained from different layers during the encoding and decoding has been provided by the DCA and CFF blocks. Furthermore, two ED blocks (Section 3.2.4) have been integrated into the architecture to pay extra attention to the edge features within the images. In addition, the PJST block (Section 3.2.5), which utilizes the advantages of both swin transformers and skip connections, has been designed and used in the encoding phase to improve the information communication and global modeling ability of the proposed architecture by capturing global features efficiently.

The proposed network adopts a pyramid architecture characterized by hierarchical multi-scale feature extraction and progressive feature reconstruction. In the encoder, features are extracted at progressively different spatial resolutions (E_1_–E_3_), enabling the network to capture contextual information at multiple scales. The decoder comprises successive up-sampling stages (D_1_–D_4_), where features are progressively reconstructed and integrated with encoder features through the DCA and CFF modules. This hierarchical organization enables the representation and fusion of both coarse-grained contextual information and fine-grained spatial details. Accordingly, the term “Pyramid Network” reflects the multi-scale, hierarchical organization of the proposed architecture.

#### 3.2.1. Encoding and Decoding Modules

The encoding module has been constructed with residual convolution blocks that include convolution processes to detect and extract local features with different spatial resolutions. These blocks have been placed at low-level (early) layers in the proposed architecture to incorporate the local feature information provided by convolutions with transformers. Recent studies have indicated that locality additions into early layers of transformers enhance feature representations [29,30].

**Figure 2:**
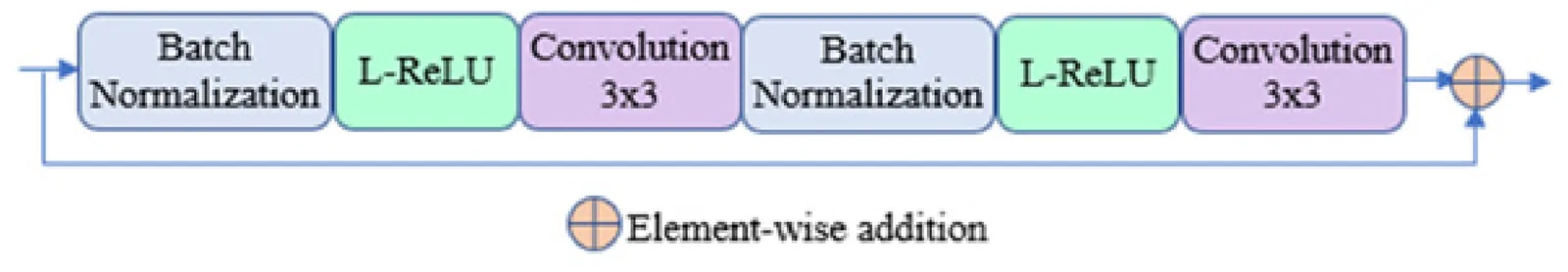
A residual convolution block.

Activation functions, which enable the learning of features through non-linear transformations in network architectures, play a crucial role, and their selection should be made carefully. They should not hamper the gradient flow, should avoid increasing computational complexity, should retain the distribution of data, and should add non-linear curvature to enhance training performance [31]. Therefore, in this work, activations in the residual convolution blocks (Figure 2) are provided by L-ReLU due to its better performance in residual networks [31].

In the encoding module, after each feature extraction stage, the sizes of the output images are 1, 1/2, and 1/4 of the size of the input. To prevent feature loss during the down-sampling operations, a convolution process (with stride 2) has been applied instead of maximum pooling. Also, the last feature map (obtained from the residual convolution block in the encoding module) has been combined with the PJST block (Section 3.2.5) through a 1 × 1 convolution to extract global features.

Like the encoding module, the decoding module has been constructed with residual convolution blocks. However, 4 layers have been used in this module. The proposed model uses 8 attention heads, embedding dimension 96, patch size 4 × 4, and window size 7.

#### 3.2.2. DCA Blocks

**Figure 3:**
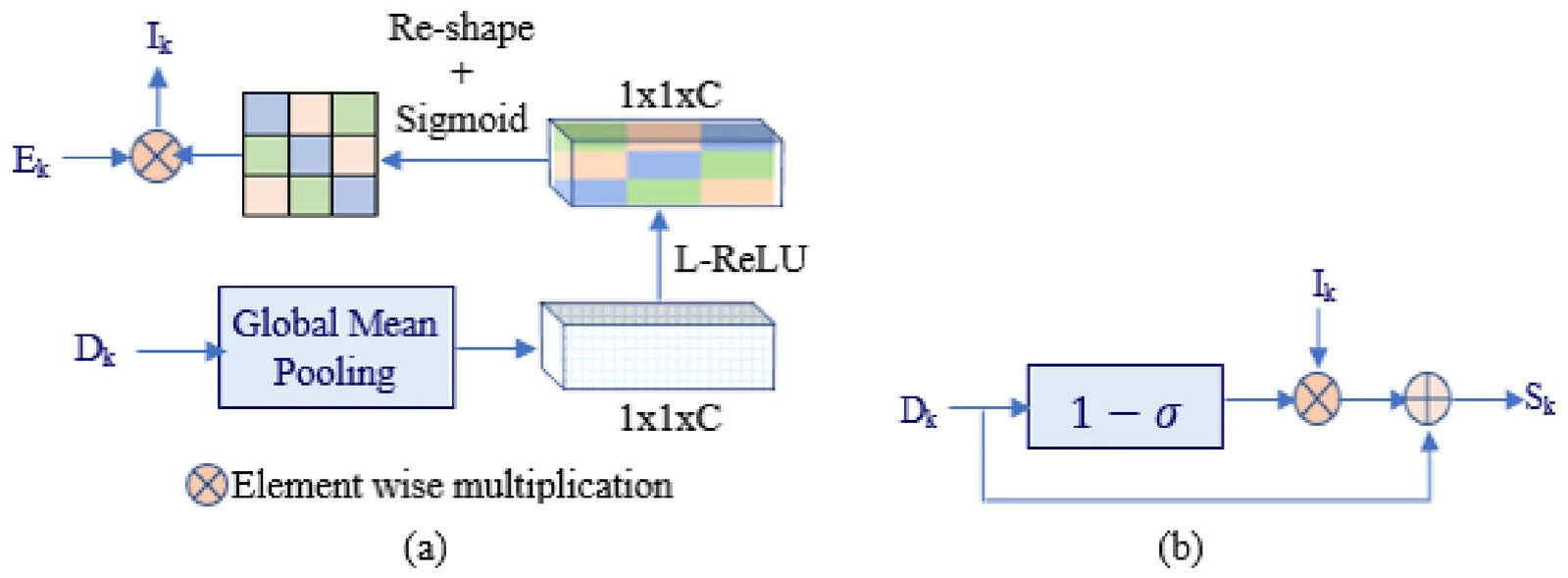
The DCA block (**a**) and the CFF block (**b**).

The DCA block (Figure 3a) has been designed so that it focuses on the channel features of the region of interest (in other words, cysts, tumors, and kidneys). This is because, in each channel, features carry distinct semantic information, and obtaining irrelevant information or information from all tissues is not required. The emphasis on those channel features of the region of interest has been provided by setting large weight values for them in the output feature channels of the convolution layers.

The inputs of the block are the inputs of the decoder’s convolution layer (in other words, *E_k_*, *k* ∊ {1, 2, 3}) and the features in the decoder after supervision (in other words, *D_k_*, *k* ∊ {1, 2, 3}). The channel attention map has been constructed using a sigmoid activation function and a linear layer after the input feature *D_k_* enters a pooling layer. In this work, global mean pooling, which is performed by computing the mean value of all the elements in the feature map, has been applied in the pooling layer to utilize its substitutability property. This is because pooling performs down-sampling operations using the same kernel size as that of the input feature maps. Through a re-shaping process, the channel attention map has been paired (matched) with the input feature (*E_k_*) space. The input feature (*E_k_*) and the channels of the weights (*F_k_*) have been multiplied to obtain the output (i.e., *I_k_, k* ∊ {1, 2, 3}) of the block.

The process in the DCA block can be formulated using the weight (*W*) and height (*H*) of the feature matrix, *D_k_* (the features in the decoder), the weights (*L* ∊ R^C×C^) of the linear layer that is used after the global mean pooling (*Pooling*(*X*) ∊ R^C×1×1^) by:

(1) $$Pooling(X)=\left(1/\left(H\times W\right)\right)\left(\sum_{r}^{H}\sum_{s}^{W}X^{c}(r,s)\right)$$

(2) $$F_{k}=\varphi \left(R_{Leaky}\left(L\cdot Pooling(D_{k})\right)\right)$$

(3) $$I_{k}=F_{k}⊗E_{k}$$

where φ refers to the sigmoid function, and $R_{Leaky}$ refers to the L-ReLU function. When information passes through the DCA block, the features that are irrelevant or not necessary are suppressed, and significant features are emphasized.

#### 3.2.3. CFF Blocks

Fuzzy and unclear boundaries, artifacts, noise, and low contrast in the images lead to difficulties in separating background and foreground regions. Therefore, the CFF block (Figure 3b) in the decoding module has been designed and used to provide feature complementation.

In this block, the first step is to generate an inverse-attention weight matrix. This matrix is generated by subtracting a weighting matrix, which is provided using the sigmoid function (*φ*), from another weighting matrix where all values are one. The next step is to generate inverse-attention features by performing an element-wise multiplication of the output of the DCA block with the inverse-attention weight matrix. The final step is re-evaluation of the region of interest area by adding the reverse-attention features to *D_k_*. These operations can be represented by the following equation, where (1 − *φ*(.)) is used to obtain the reverse masks, and *φ*(.) refers to the sigmoid function:

(4) $$S_{k}=I_{k}⊗\left(1-\varphi (D_{k})\right)+D_{k}$$

#### 3.2.4. ED Blocks

Two ED blocks have been integrated into the decoding module to accurately obtain edge features since it is difficult to detect the region-of-interest area if edges are unclear and the area includes inhomogeneous gray values.

In the ED blocks, a 1 × 1 convolution operation has been applied to create the segmentation map (S_map_(i)). After that, the pixel range has been expanded using a maximum-pooling operation, and the feature map (F_map_(I)) has been generated. Finally, the boundary map is obtained by subtracting the feature map (F_map_(I)) from the segmentation map (S_map_(i)) using element-wise subtraction.

#### 3.2.5. The PJST Block

**Figure 4:**
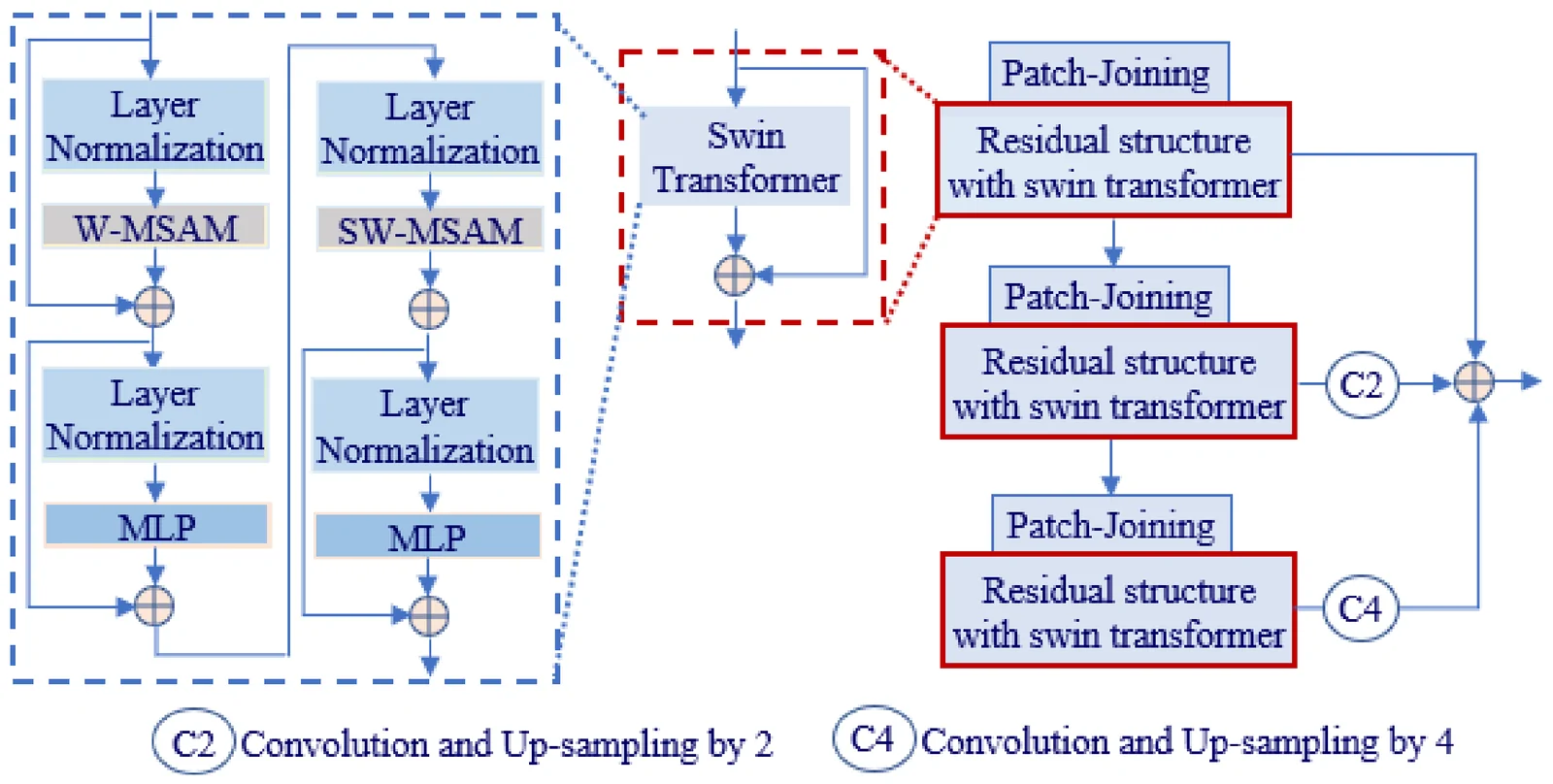
The PJST block.

The PJST block has been constructed with 3 layers, each of which includes a patch-joining stage and a residual structure with a swin transformer (Figure 4). The input and output of the transformer are linked with residual (skip) connections. These connections allow the aggregation of features coming from different levels.

The swin transformer uses a hierarchical construction method in CNNs and is a hierarchical transformer [8]. The standard vision transformer has strong modeling capabilities. Visual priors make the standard transformer friendly to a variety of visual tasks.

The strengths of the standard transformer and visual priors are combined in the swin transformer by introducing significant visual priors (i.e., translation invariance, spatial locality, and hierarchy) into the standard transformer.

The swin transformer introduces a local self-attention mechanism (SAM) within non-overlapping local windows to adequately capture local feature details. In other words, instead of the Multi-head SAM (MSAM) that performs self-attention across the entire image in the standard transformer, a Window-MSAM (W-MSAM) is used in the swin transformer. These windows, including patches sized *M* × *M* (*M* is 7 by default), operate independently, which leads to a loss of inter-window connectivity due to their fixed separations. For this reason, a Shifted Window-MSAM (SW-MSAM) is also used in the swin transformer for cross-window interaction.

The SW-MSAM and W-MSAM are executed within each window rather than across the entire image space, alternating to maintain connectivity and enable information exchange (interaction) among windows. However, this alternating process leads to limitations in the application of local window-based attention and reduces global modeling capabilities. Therefore, in this work, the PJST block has been developed to improve the information communication and global modeling capability of the proposed architecture.

In the PJST block, where patches are joined via element-wise addition, joined patches are inputs of the transformers. The joined patches and the information provided by the transformers are combined by an element-wise addition to maintain the integrity of features coming from different layers. Also, the outputs of this operation are used as inputs for the succeeding patch-joining process. To ensure spatial alignment of the output features from the top layer, a 1 × 1 convolution and an up-sampling process are applied to the features obtained from the last and the middle layers. After the up-sampling operations (by 2 and 4 to the features from the last and middle layers, respectively), feature aggregation is performed. For this reason, element-wise addition is applied with the up-sampled features and the output features from the top layer.

#### 3.2.6. Loss Function

Some studies demonstrated that the feature distinguishability of the cross-entropy loss function may be ineffective [32,33]. Similarly, the Dice loss function can be ineffective in the discrimination of complex features and fuzzy borders of the kidneys, tumors, and cysts. Thus, a weighted hybrid (cross-entropy and Dice) loss function, combining Dice and cross-entropy functions, was implemented in this study to provide effective feature discrimination and improve the training stage by combining shapes and probability distributions. The hybrid loss function is defined using $\lambda_{1}$ and $\lambda_{2}$ weight values (which have been experimentally set as $\lambda_{1}=\lambda_{2}=$ 0.5) by:

(5) $$L=\lambda_{1}L_{CE}+\lambda_{2}L_{Dice}$$

where the terms *L_CE_* and *L_Dice_* refer to the cross-entropy and Dice functions, respectively. The *L_Dice_* function is computed by $L_{Dice}=1-\left(2\sum_{j=1}^{M}\left(y_{jc}{\hat{y}}_{jc}\right)/\sum_{j=1}^{M}\left(y_{jc}+{\hat{y}}_{jc}\right)\right)$, and the *L_CE_* function is computed by $L_{CE}=\sum_{j=1}^{M}\left[y_{jc}\log{\hat{y}}_{jc}+(1-y_{jc})\log(1-{\hat{y}}_{jc})\right]$, where the term c represents the total number of classes, which is 4 (background, cyst, tumor, and kidney) in our method. The term *y_jc_* refers to the *j*-th ground truth pixel of class *c*, while ${\hat{y}}_{jc}$ denotes the corresponding predicted probability. *M* denotes the total number of pixels of the input image.

The predicted logical values have been converted into probabilistic values using the softmax function. Subsequently, the probabilistic maps have been transformed into segmentation masks based on the maximum probability class.

## 4. Results

This section presents the qualitative and quantitative evaluation results (Section 4.1), describes the implementation details (Section 4.2), and reports the ablation study results (Section 4.3).

### 4.1. Detailed Results

**Figure 5:**
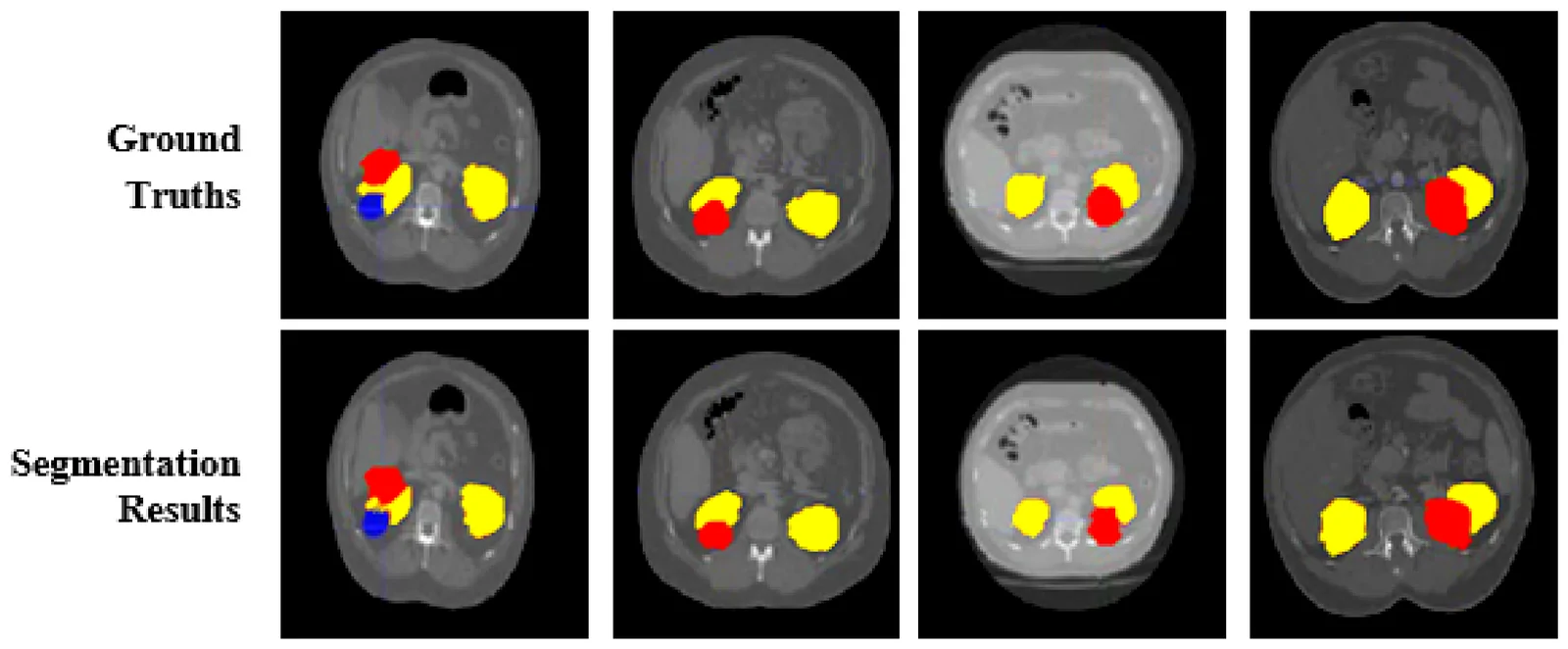
The results obtained using images from the KiTS-2023 database [27] and their corresponding ground truths (kidneys, tumors, and cysts are represented in yellow, red, and blue, respectively).

The results of the proposed approach have been analyzed both quantitatively and qualitatively. Example images showing the ground truths and the results obtained automatically from the proposed approach using images from the KiTS-2023 database [27] have been presented in Figure 5.

**Figure 6:**
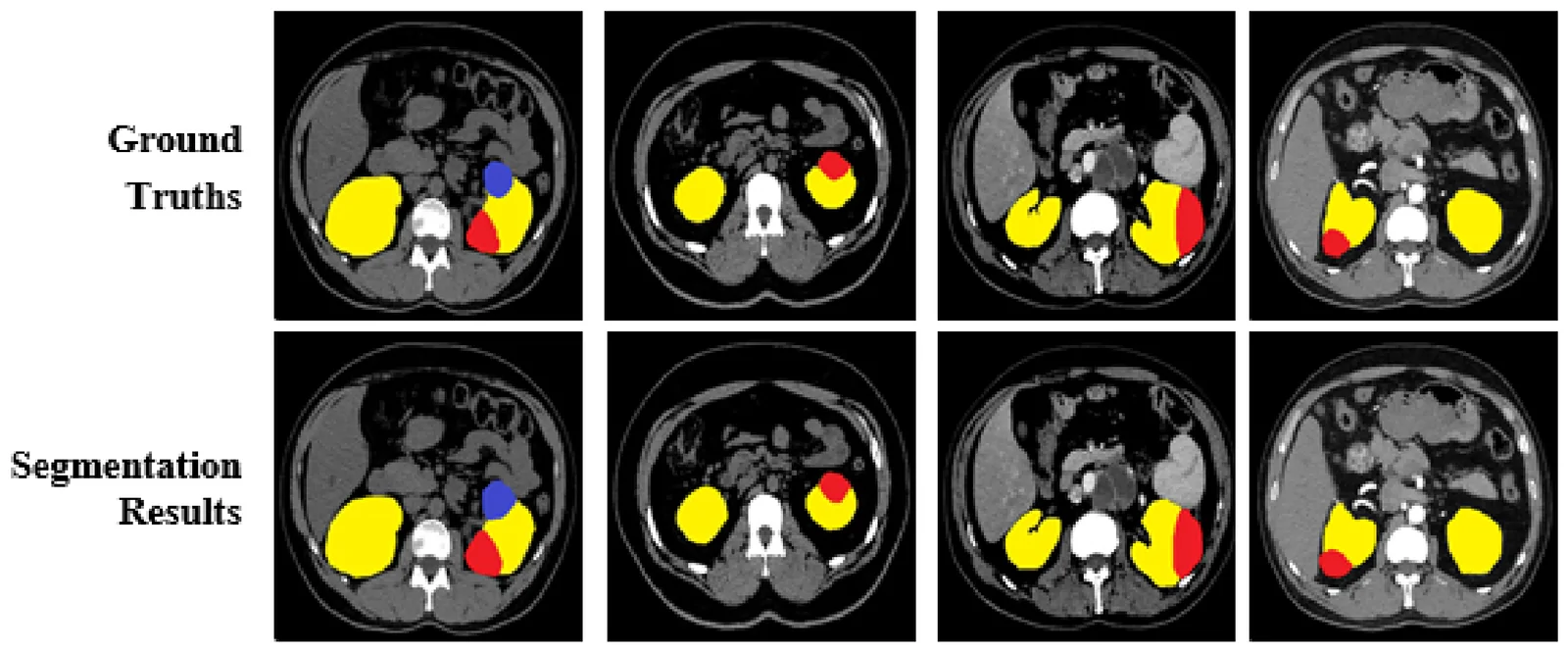
The results obtained using images from the Kaggle website [28] and their corresponding ground truths (kidneys, tumors, and cysts are represented in yellow, red, and blue, respectively).

Visualizations of the images (showing kidneys, tumors, and cysts as yellow, red, and blue, respectively) have been generated using the ITK-snap (version 4.2.0) tool, which is open-source software [34]. Also, example images showing the segmentation results obtained using images from the Kaggle website [28] and their corresponding ground truths are presented in Figure 6.

**Figure 7:**
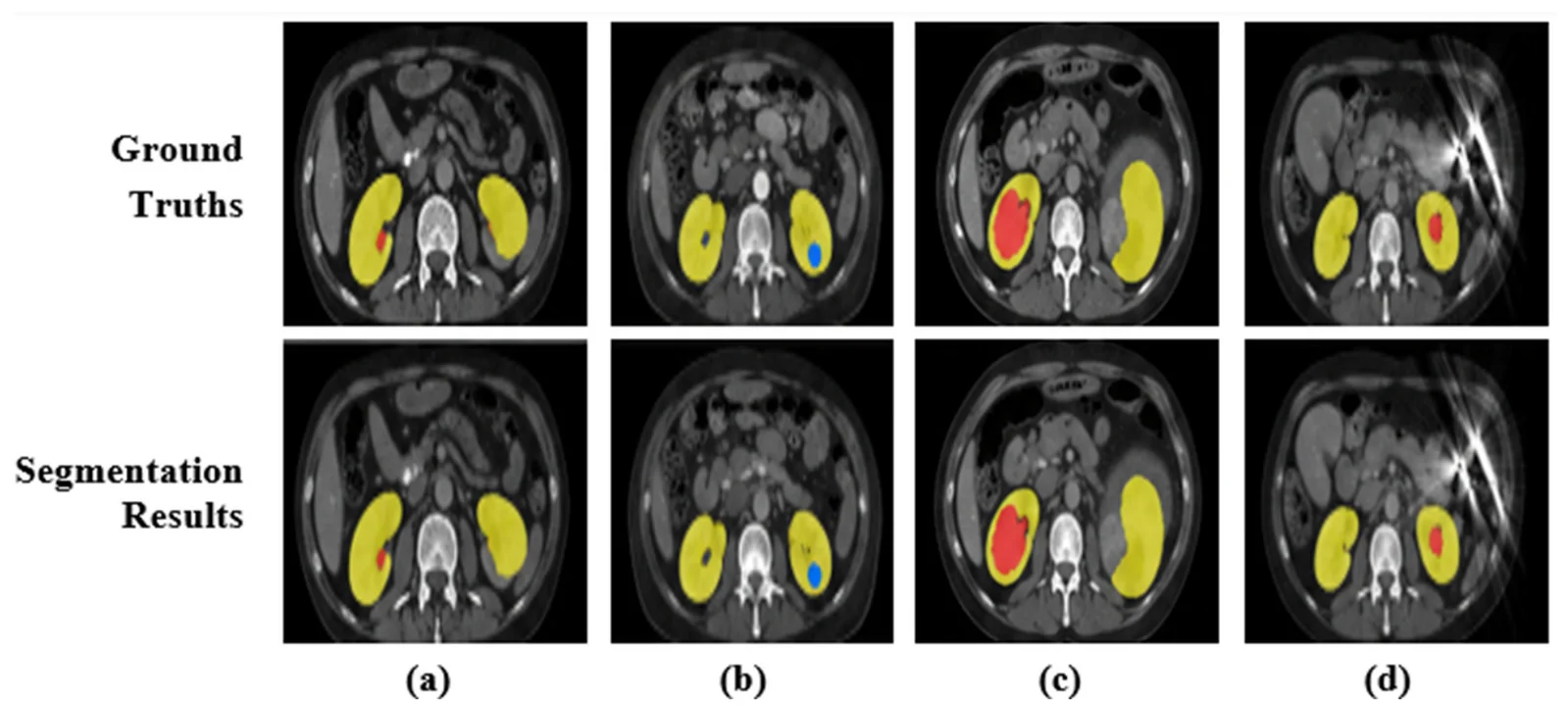
The results for challenging cases, including small tumors (**a**), small cysts (**b**), blurred boundaries (**c**), and imaging artifacts (**d**) (kidneys, tumors, and cysts are represented in yellow, red, and blue, respectively).

Figure 7 shows segmentation results for challenging cases, including small tumors (a), small cysts (b), blurred boundaries (c), and imaging artifacts (d). For each case, ground-truth annotations and segmentation results obtained using the proposed method are presented. The selected cases illustrate the performance of the proposed method under challenging imaging conditions and its ability to delineate different anatomical structures and pathological regions.

Quantitative analyses have been performed with the commonly used evaluation metric, which is known as the DSC [35]. It is also known as the F1-score and is computed based on the classifications of the pixels in a segmented image. The following terms are used in its computation:

- True Positives (TP): This term refers to the pixels correctly identified as part of a kidney, tumor, or cyst, respectively.
- True Negatives (TN): This term refers to the pixels correctly identified as not part of a kidney, tumor, or cyst, respectively.
- False Positives (FP): This term refers to the pixels wrongly identified as part of a kidney, tumor, or cyst, respectively.
- False Negatives (FN): This term refers to the pixels wrongly identified as not part of a kidney, tumor, or cyst, respectively.

The metric is obtained by computing the ratio of twice the intersection of the pixels recognized as kidney, tumor, or cyst in both the segmentation results and the ground truths over the sum of these pixels. Therefore, its definition is written based on the segmentation result (S) and ground truth (G) as:

(6) $$\begin{array}{l} \mathrm{DSC}=\frac{2\left|G\cap S\right|}{\left|G\right|+\left|S\right|}\\ =\frac{2\left|G\cap S\right|}{\left|S\backslash G\right|+\left|G\cap S\right|+\left|G\backslash S\right|+\left|G\cap S\right|}=\frac{2\left|G\cap S\right|}{\left|S\backslash G\right|+\left|G\backslash S\right|+2\left|G\cap S\right|}=\frac{2\mathrm{TP}}{2\mathrm{TP}+\mathrm{FN}+\mathrm{FP}} \end{array}$$

The DSC values are in [0, 1], and a higher DSC indicates a higher performance (i.e., a better match between the ground truth and segmentation results). Also, NSD [36] has been employed. Unlike DSC, a surface-based metric measuring the overlap between two shapes/objects, the NSD is a boundary-based evaluation metric measuring the overlap between two boundaries. In other words, this metric is utilized to assess the agreement of boundaries between segmentation results (within a specified tolerance, which is a user-defined threshold) and expert annotation masks. It is calculated by:

(7) $$\mathrm{NSD}(G,S)=\left(\left|\partial S\cap B_{\partial G}^{\tau }\right|+\left|\partial G\cap B_{\partial S}^{\tau }\right|\right)/\left(\left|\partial S\right|+\left|\partial G\right|\right)$$

where the terms $B_{\partial S}^{\tau }$ and $B_{\partial G}^{\tau }$ refer to the border regions of the segmentation surfaces at tolerance τ (which is 2 in this work) and the expert annotation masks, respectively.

In addition, the performance of the proposed method and the state-of-the-art methods has been compared. To ensure fair comparisons, these methods have been applied to the same datasets, and their results have been evaluated based on the same evaluation metrics. The results of the methods using images from the KiTS-2023 database, in terms of the DSC and NSD metrics, are presented in Table 3 and Table 4, respectively.

The results of the methods using images from the Kaggle website, in terms of the DSC and NSD metrics, are presented in Table 5 and Table 6, respectively.

Experimental results obtained from the methods using images provided from both the KiTS-2023 database and the Kaggle website, in terms of the DSC and NSD metrics, are presented in Table 7 and Table 8, respectively.

Also, statistical significance was computed via a paired *t*-test across test folds. All improvements of ≥1.0% DSC were statistically significant (*p* < 0.05). In addition to the paired *t*-test, a non-parametric Wilcoxon signed-rank test was performed on the fold-wise DSC results to account for potential deviations from normality. The Wilcoxon test confirmed the statistical significance of all performance improvements of ≥1.0% DSC (*p* < 0.05), yielding conclusions consistent with those obtained using the paired *t*-test.

### 4.2. Implementation Details

For all implementations in this work, the number of epochs and the learning rate values have been set to 200 and 0.0001, respectively. Stochastic gradient descent (with momentum = 0.9) has been applied; weight decay and batch size values have been set to 0.0001 and 8, respectively. All methods have been implemented using Pytorch 3.9.7 on a system with an NVIDIA GTX-1070 graphics processing unit, 8 gigabytes of processing unit memory, 24 gigabytes of read-access memory, and an Intel Core-i7-7700 processor running at 3.60 gigahertz.

### 4.3. Ablation Studies

The effectiveness of each block has been investigated. To see the effect of the DCA block, the proposed network has been applied with and without the DCA block using images from the KiTS-2023 database. Then, those networks have been trained and tested using the same datasets. It has been observed that, when these blocks have been integrated, the average performance for the segmentation of kidneys, tumors, and cysts has improved by 22.94%, 26.71%, and 21.95%, respectively (Table 9).

Similarly, to see the effect of the CFF block, the proposed network has been applied with and without the CFF block; then those networks have been trained and tested using the same datasets. It has been observed that, when these blocks have been added, the average performance for the segmentation of kidneys, tumors, and cysts has improved by 1.57%, 1.49%, and 1.46%, respectively (Table 9).

Also, the network with and without the four blocks (DCA, CFF, ED, and PJST) has been trained and tested using the same datasets. When these four blocks have been added, the average performance for the segmentation of kidneys, tumors, and cysts has improved by 27.09%, 30.85%, and 25.91%, respectively (Table 9).

To assess the robustness and consistency of the observed performance improvements, all ablation experiments were conducted using a five-fold evaluation protocol. The results are reported as mean ± standard deviation across the five folds for each evaluation metric (Table 9). This protocol allows the variability in segmentation performance across different data partitions to be quantified and provides a more comprehensive assessment of the individual contributions of the proposed modules.

Additionally, the methods were evaluated in terms of computational efficiency. As longer training times indicate higher computational complexity, training time values were considered in this study. Therefore, the training time required by each method with the images from the KiTS-2023 dataset was recorded for each of the five folds. The reported values in Table 10 represent the average training time across the five folds.

To examine the impact of the hybrid loss function, the proposed model has been implemented with commonly used loss functions (i.e., weighted cross-entropy with experimentally set optimal weights (0.5), Dice, and focal loss functions) separately. The results obtained from these methods using images provided from both the KiTS-2023 database and the Kaggle website, in terms of the DSC, are presented in Table 11.

## 5. Discussion

In general, the experimental results obtained using images from the KiTS-2023 database (Table 3 and Table 4) are higher than the results obtained using images from the Kaggle website (Table 5 and Table 6). This is because the images in the KiTS-2023 database are more challenging than the images provided on the Kaggle website.

Also, according to the quantitative analyses, the performance of the applied methods is lower when measured by NSD (Table 4, Table 6 and Table 8) metrics compared to DSC metrics (Table 3, Table 5 and Table 7). This indicates that they are not always efficient in detecting boundaries of the kidneys, tumors, or cysts, mainly due to their unclear edges and complex shapes, particularly when their sizes are small.

Transformers and CNNs are effective in the segmentation of kidneys, tumors, and cysts from CT scans. CNNs can perform the extraction of local features using various pooling and convolution operations. However, a CNN architecture focuses on pixel-wise features and ignores global information. Therefore, among the methods in the current literature, pure CNN-based models [15,16,17] exhibit lower performance than CNNs incorporated with attention mechanisms [19,20,21,22] (Table 3, Table 4, Table 5, Table 6, Table 7 and Table 8).

Transformers have the ability to extract global features with the help of their receptive fields. However, they are bad at the extraction of local information and details. Therefore, a network consisting solely of a CNN or transformer is not efficient in the segmentation of kidneys, their tumors, and cysts for many reasons, such as unclear edges, inhomogeneous intensities, artifacts, atypical shapes, and similar intensity values with other tissues.

The methods in [19,20,21,22] include both a transformer and a CNN, thereby producing more effective results than the others (Table 3, Table 4, Table 5, Table 6, Table 7 and Table 8). On the other hand, their ability to fully utilize convolution operations and transformers is not effective for segmenting kidneys, tumors and cysts. Therefore, their performance is lower than the proposed model, which has a pyramid-shaped structure and a more effective design for fully utilizing the advantages of both CNNs and transformers (Table 3, Table 4, Table 5, Table 6, Table 7 and Table 8).

Also, the segmentation performance of the proposed hybrid model has been enhanced by integrating the PJST, DCA, CFF, and ED blocks. The ablation results in Table 9 demonstrate that each component of the proposed hybrid architecture contributes incrementally to segmentation accuracy. These findings confirm that the proposed blocks provide complementary benefits and are jointly responsible for the observed improvement. The PJST block utilizes the advantages of both swin transformers and skip connections, improving information communication and the global modeling ability of the proposed architecture by capturing global features efficiently. The channel guidance (using the DCA blocks) and spatial guidance (using the CFF blocks) have further improved the segmentation performance. This is because the DCA block, which uses outputs of the PJST, enables close attention to the regions that are to be segmented and sends required features to other steps, while also suppressing unnecessary information. The guidance in the CFF block enables close attention to features in the regions with weak responses (i.e., weak features) by adding information from other steps. In this way, the detection of underestimated areas is improved.

Although the DCA and CFF modules are inspired by prior attention structures (CBAM and SE-Net), they have key differences, as presented in Table 12.

The commonly used activation function in the literature is ReLU, which can cause dying neurons whenever large gradients flow into them [25]. Additionally, the effect known as bias-shifting occurs if a unit is activated by ReLU. Furthermore, learning becomes slow if the calculated mean value deviates significantly from zero [26]. In this work, these problems have been overcome by using L-ReLU for activations.

The proposed architecture performs better than current state-of-the-art approaches. The improvement in segmentation accuracy has been achieved through the following: (i) It has both local feature extraction ability with a CNN and global feature extraction ability with a transformer. (ii) The L-ReLU activates neurons without leading to any dying neurons or biasing effects. (iii) The PJST block enhances information communication and global modeling ability of the architecture. (iv) The DCA blocks highlight the kidney, tumor, and cyst, and suppress unnecessary information by routing the channel features to the decoder section. (v) The CFF blocks merge the encoder’s multi-scale features effectively and provide complementation of the features coming from the decoder. (vi) The ED blocks further enhance the detection of the edges.

To investigate the effect of different loss functions on segmentation performance, ablation studies were conducted using weighted cross-entropy, Dice loss, focal loss, and the proposed hybrid loss (Table 11). These loss functions have different optimization characteristics. Cross-entropy measures the discrepancy between the ground-truth labels and predicted class probabilities, whereas Dice loss directly optimizes the overlap between predicted and ground-truth regions. Focal loss places greater emphasis on hard-to-classify pixels by reducing the contribution of well-classified pixels. The hybrid loss combines the complementary characteristics of cross-entropy and Dice loss, enabling the model to optimize both pixel-wise classification accuracy and region-level overlap. The experimental results presented in Table 11 demonstrate that the proposed hybrid loss achieved higher DSC values than the individual loss functions evaluated in this study. Specifically, compared with standard cross-entropy, the hybrid loss improved the DSC for kidneys, tumors, and cysts by 11.08 ± 0.06%, 10.79 ± 0.01%, and 2.84 ± 0.18%, respectively. These findings suggest that combining cross-entropy and Dice loss can improve segmentation performance across different anatomical structures, particularly in the evaluated multi-class segmentation setting. The observed improvements support the use of the hybrid loss in the proposed architecture, although further experiments would be required to establish its generalizability across different datasets and class distributions.

The training time of the investigated methods, reported in Table 10, should be considered alongside their segmentation performance presented in Table 4, Table 5, Table 6, Table 7, Table 8 and Table 9. These tables collectively provide a basis for assessing the relationship between segmentation accuracy and training time for kidneys, tumors, and cysts. This analysis considers the segmentation performance in conjunction with the average training time across the five folds. This comparison provides further insight into the computational cost associated with the performance achieved by the proposed architecture. Therefore, a comparative analysis of segmentation performance and training time was conducted to further investigate the performance-efficiency trade-off of the proposed method. As the Asymmetric-HybridModel [20] achieves higher performance than the other methods, a comparative analysis of its NSD scores and training times with those of the proposed method can be presented here. Compared with the Asymmetric-HybridModel [20], the proposed method achieved higher NSD scores for all three segmentation targets, with improvements of 6.82%, 10.58%, and 5.23% for kidneys, tumors, and cysts, respectively (Table 8). These improvements were obtained at the expense of an additional 13.4 min of training time (Table 10). Notably, the largest improvement was observed in tumor segmentation, indicating a particularly substantial gain in boundary delineation for this target. Also, the experiments showed that the RenalSegNet [17] requires the shortest training time (43.3 min) (Table 10); however, its performance is lower than that of the methods in [18,19,20,21,22] and the proposed method (Table 3, Table 4, Table 5, Table 6, Table 7 and Table 8). Similarly, the model combining DeepLabV3 and Xception65 [15] requires less training time (50.0 min) than others (Table 10) but still underperforms compared to the methods in [18,19,20,21,22] and the proposed method (Table 3, Table 4, Table 5, Table 6, Table 7 and Table 8). The proposed method requires 53.3 min for training, making it the most effective approach among all methods regarding both computational complexity and accuracy. Overall, these results demonstrate that the proposed method achieves improved segmentation performance across all three targets with a moderate increase in training time. This trade-off should be considered in the context of the intended application and the relative importance of segmentation accuracy and computational cost. It is important to note that the training times in Table 10 are affected by the experimental setup and may vary based on implementation and hardware factors.

To provide a more comprehensive evaluation of the proposed method, additional qualitative experiments were conducted on challenging CT images. These cases were selected to investigate the model’s performance under difficult imaging conditions, including small tumors and cysts, blurred boundaries, and imaging artifacts. Figure 7 presents a comparison of the ground-truth annotations and the segmentation predictions generated by the proposed method. The segmentation of small tumors and cysts is particularly challenging because these structures occupy relatively few pixels, making their accurate localization and boundary delineation more sensitive to small prediction errors. This issue is especially relevant to cyst segmentation, for which the segmentation performance is lower than that of the kidney and tumor classes. The qualitative examples in Figure 7 illustrate the challenges associated with delineating small lesions and demonstrate the importance of preserving fine-grained spatial information during segmentation. Furthermore, blurred boundaries and low contrast may reduce the separability of different tissue classes. Imaging artifacts may introduce additional ambiguities by altering local intensity patterns and obscuring anatomical structures. These factors can contribute to inaccurate boundary localization or the inclusion of surrounding tissues in the predicted masks. Overall, the challenging examples provide a complementary assessment of the proposed method beyond the quantitative metrics and representative successful cases. They highlight the difficulties associated with segmenting small and poorly delineated renal lesions and provide further insight into the factors that may contribute to the comparatively lower performance in cyst segmentation.

The proposed four blocks (PJST, DCA, CFF, and ED) introduce additional computational operations and learnable parameters, thereby contributing to the overall computational complexity of the model. In particular, the computational cost depends on the operations performed within each block and the dimensions of the feature maps processed. Although these blocks increase the architectural complexity, they are designed to improve the extraction, integration, and refinement of complementary features for multi-class segmentation. Therefore, the computational overhead should be considered alongside the segmentation performance improvements achieved by the proposed architecture. A comprehensive assessment of this trade-off requires considering both the model’s parameter count and its computational cost.

In order to further contextualize the performance of the proposed method, a horizontal comparison with other medical image segmentation tasks, such as liver and brain tumor segmentation, can be considered. Compared to liver segmentation, which generally involves a relatively large organ with smoother boundaries and more stable anatomical location, kidney segmentation is more challenging due to higher inter-patient variability, smaller organ size, and frequent intensity similarity with surrounding tissues. These challenges are further amplified by the presence of kidney tumors and cysts, which often exhibit heterogeneous textures, unclear boundaries, and variable morphologies. In contrast, brain tumor segmentation is typically performed on multi-modal MRI data, where complementary imaging sequences provide rich contrast information that facilitates lesion delineation. However, kidney segmentation from CT images relies primarily on single-modality intensity information and is therefore more sensitive to noise, artifacts, and low soft-tissue contrast. This makes the accurate detection of kidney boundaries and associated lesions considerably more difficult. Within this context, the proposed pyramid-based hybrid architecture demonstrates strong adaptability across different medical image segmentation scenarios. The integration of CNNs and transformers enables effective local feature extraction while simultaneously capturing long-range dependencies, which is beneficial for organs with complex spatial structures. The PJST block enhances global modeling ability beyond conventional window-based attention mechanisms, making it suitable for handling organs and lesions with variable shapes and unpredictable locations. Moreover, the DCA and CFF blocks provide complementary channel-wise and spatial guidance that improve feature discrimination in regions with weak responses, a characteristic commonly encountered in kidney, liver, and brain tumor segmentation tasks. The ED blocks further strengthen boundary awareness, which is critical for accurate segmentation in cases with fuzzy or irregular edges. These observations suggest that although kidney, tumor, and cyst segmentation from CT images presents unique challenges compared to other medical image segmentation problems, the proposed architecture demonstrates promising generalization capability across the two publicly available datasets evaluated in this study. Its design principles allow it to be effectively extended to other organ and lesion segmentation tasks with minimal architectural modifications.

The proposed method has the potential to support medical experts by providing quantitative and objective segmentation results that may contribute to disease assessment, severity estimation, and surgical and treatment planning. However, its clinical utility and reliability in these applications require further investigation and clinical validation. Its generalizability should not be considered established across all clinical settings. The two datasets may not fully represent the variability in imaging protocols, scanners, patient populations, and clinical practices encountered in real-world applications. Therefore, further evaluation on additional datasets collected from different institutions and using diverse imaging protocols is required to more comprehensively assess the robustness and generalization capability of the proposed method.

Although the proposed method achieves high overall performance, a class-specific error analysis reveals that cyst segmentation is more challenging than that of kidney and tumor segmentations. This can be attributed to several characteristics of cysts in CT images. First, cysts may exhibit indistinct boundaries and low contrast with the surrounding renal parenchyma, making it difficult for the network to accurately identify their spatial extent. This ambiguity can result in boundary-related errors, including the omission of peripheral cyst regions (FN) or the inclusion of adjacent kidney tissue (FP). Second, cysts exhibit considerable variations in size, shape, and spatial distribution. Such heterogeneity increases the difficulty of learning consistent class-specific representations, particularly when cysts have irregular shapes or occupy relatively small image regions. Furthermore, partial-volume effects and local intensity variations may obscure cyst boundaries, further complicating the discrimination between cysts and surrounding tissues. In contrast, kidney segmentation benefits from the relatively large spatial extent and more coherent anatomical structure of the kidneys, which provide stronger contextual information for delineation. Although tumors may exhibit substantial morphological variability and indistinct boundaries, their segmentation performance is higher than that of cysts in the evaluated dataset. This suggests that cyst segmentation presents additional challenges related to their appearance, boundary characteristics, and morphological variability. The lower cyst DSC is also consistent with the reduced NSD, indicating that cyst segmentation performance is affected not only in terms of volumetric overlap but also in terms of boundary agreement.

**Figure 8:**
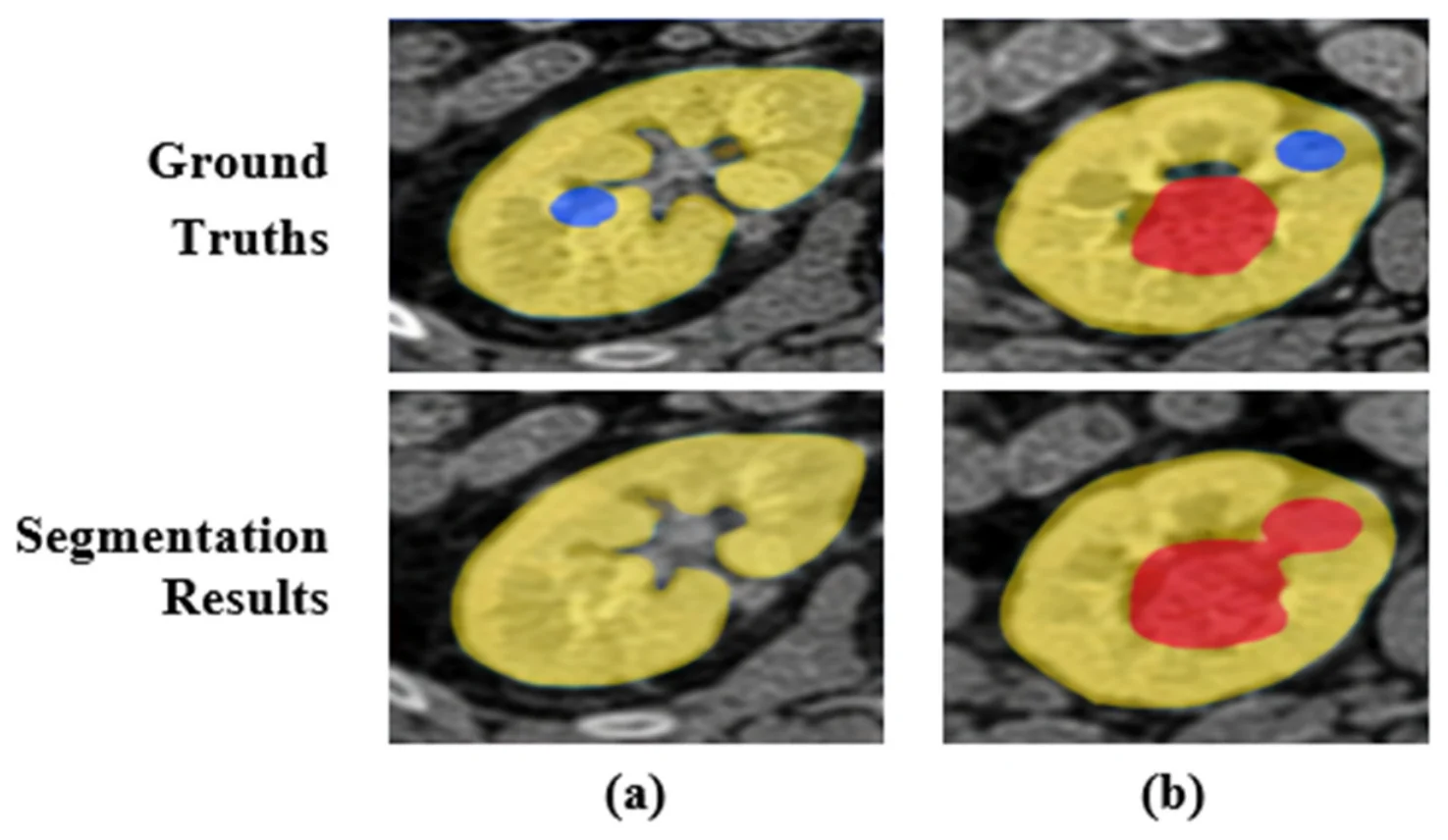
A missed small-sized cyst in the predicted segmentation (**a**), segmentation boundary inaccuracies and cyst–tumor confusion (**b**) (kidneys, tumors, and cysts are represented in yellow, red, and blue, respectively).

To further improve cyst segmentation performance, several future research directions can be considered. For instance, incorporating stronger shape priors may help enforce structural consistency and reduce false detections. Additionally, introducing more advanced multi-scale or hierarchical attention mechanisms could enhance the representation of subtle cyst features across different spatial resolutions. Exploring uncertainty-aware learning strategies may also reduce confusion between cysts and tumors. Representative failure cases of the proposed method in kidney, tumor, and cyst segmentation are presented in Figure 8. These cases include a missed small cyst in the predicted segmentation (Figure 8a) and segmentation boundary inaccuracies and cyst–tumor confusion (Figure 8b). For each case, the ground-truth annotations and the predictions generated by the proposed method are displayed in zoomed-in regions to highlight segmentation discrepancies and improve their visibility.

## 6. Conclusions

Inspired by recent studies, a novel pyramid-shaped network has been designed in this work for image segmentation by using CNN and vision transformer structures. In the proposed hybrid architecture, CNNs are used to extract semantic features, while transformers extract global features and create dependencies on the semantic features. The information communication and global modeling ability of the architecture have been improved by the PJST block. Additionally, the DCA block has been designed and integrated into the architecture to focus more on the regions that need to be segmented and send the required features to other steps while suppressing unnecessary information. The DCA block, which uses outputs from the PJST, guides the channel features from the CNN to be sent through the decoder. Furthermore, the CFF blocks have been employed to merge the encoder’s multi-scale features effectively and provide complementation of the features coming from the decoder. The ED blocks have also been used to enhance the detection of the edges. The proposed architecture has been trained and tested to investigate its effect on the segmentation of kidneys, tumors, and cysts.

It has been observed that the DCA blocks efficiently improve convolutional layers with their channel-attention mechanisms to focus on kidneys, tumors, and cysts in the images. Also, the CFF block can focus attention on accurate positions and provide the required information and a rich representation of features. The CFF and ED blocks boost the segmentation performance by effectively merging and complementing features and enhancing edge detection. The combination of DCA, CFF, ED, and PJST blocks enables more efficient interactions among features at diverse scales, thereby enhancing segmentation performance through more effective combinations of feature representations (Table 9).

It has been observed from the experiments that the extraction and learning of significant feature information, fusion of multi-scale features, and their propagation across layers is achieved efficiently by the proposed network. It provides high-performance segmentations of kidneys, tumors, and cysts (Table 7 and Table 8).

Also, in this work, the state-of-the-art methods have undergone training and testing using identical datasets for fair comparisons with the proposed method. Average segmentation results in terms of DSC have indicated the superiority of the proposed approach in segmenting kidneys (98.35 ± 0.65%), tumors (94.88 ± 0.57%), and cysts (81.99 ± 0.45%) (Table 7). Similarly, average segmentation results in terms of NSD have indicated the superiority of the proposed approach in segmenting kidneys (97.62 ± 0.55%), tumors (93.84 ± 0.50%), and cysts (80.93 ± 0.40%) (Table 8).

The proposed method is a promising approach. It shows potential for supporting medical experts by providing quantitative and objective segmentation results that may contribute to disease assessment and surgical or treatment planning. It can eliminate the problems caused by subjectivity and conventional methods that depend on visual examinations of images. In addition, it can alleviate the experts’ workload and enable them to allocate time for other operations and tasks. Also, the proposed approach can be beneficial for researchers interested in analyzing CT images and developing novel segmentation methods.

## Figures and Tables

**Table 1:** The methods proposed for segmentation of kidneys, tumors, and cysts from CT scans.

Method, Year	Datasets	Architecture	Segmentation for			Limitation/Disadvantage	Mean Results (%)
			Kidney	Tumor	Cyst		
DeepLabV3 and Xception65 [15], 2025	Private	A combination of DeepLabV3 and Xception65 models	√	─	─	Its performance may decrease when tested with datasets with more numbers and variations in images, as the ReLU causes dying neurons. Its effect on segmenting tumors and cysts is unclear.	DSC: 95.00 IoU: 92.00
PSTUDA [16], 2025	KiTS-2019	A CNN model with a shared generator and a discriminator	√	√	─	It may not always be effective, as aligning multi-modality images is needed, and missing modalities in the real world often occur. Also, its effect on cyst segmentation is unclear.	For kidneys; DSC: 85.91 For tumors; DSC: 79.17
RenalSegNet [17], 2025	Kidney Parsing 2022 (KiPA22) [17]	A CNN with an encoder–decoder structure including squeeze-and-excitation-based feature extraction	√	√	─	It causes over-segmentation when segmenting small tumors. The ReLU may diminish its performance due to dying neurons when it is tested using datasets with more variations.	For kidneys; DSC: 95.89 IoU: 92.12 For tumors; DSC: 91.68 IoU: 84.69
Dual-stage segmentation [18], 2025	KiTS-2023	SwinUNETR and a UNet with an attention mechanism to segment kidneys and tumors, respectively	√	√	─	The UNet model, which mainly depends on convolution operations, can be ineffective at capturing desired features, causing low performance in segmenting tumors. Its effect on cyst segmentation is unclear.	For kidneys; DSC: 97.00 For tumors; DSC: 91.00
Swin-IBNet [19], 2025	Synapse [19]	Fusion of a CNN with swin transformer	√	─	─	It may be ineffective due to the swin transformer, whose global modeling ability is less than that of the original transformer. The ReLU may cause poor performance. It was not tested for segmenting kidney tumors and cysts.	DSC: 84.38
Asymmetric-HybridModel [20], 2025	Chaos [20]	A model combining a CNN-transformer feature fusion module with a cross-modality graph structure	√	─	─	It is ineffective with incomplete image modalities, as aligning multi-modal images is needed, and missing modalities in real-world setting often occur. It was not tested for segmenting kidney tumors and cysts.	DSC: 92.14
SG-UNet [21], 2025	Synapse [19]	Fusion of a U-Net model with a self-guided transformer structure	√	─	─	The ReLU may cause poor performance when it is tested with more diverse images. It was not tested for segmenting kidney tumors and cysts.	DSC: 86.03
DPEGNet [22], 2025	Synapse [19]	A dual-path transformer with a group cross-aggregation structure	√	─	─	It suffers from over-segmentation when intricate boundaries exist. It was not tested for segmenting kidney tumors and cysts.	DSC: 86.03

**Table 2:** The split procedure employed in this study.

Dataset	Total Images	Number of Images		
		For Test	For Training	For Validation
KiTS-2023	590	110	384	96
Kaggle	890	210	544	136

**Table 3:** DSC results (in %) using images from the KiTS-2023 database.

Method	DSC for Kidneys	DSC for Tumors	DSC for Cysts
PSTUDA [16]	83.97 ± 1.48	78.56 ± 1.57	65.71 ± 1.64
DeepLabV3 and Xception65 [15]	84.78 ± 1.96	78.80 ± 1.99	66.58 ± 1.97
RenalSegNet [17]	84.81 ± 1.74	80.36 ± 1.96	68.99 ± 1.94
Dual-stage segmentation [18]	84.92 ± 1.51	80.87 ± 1.93	70.17 ± 1.91
Swin-IBNet [19]	85.72 ± 1.19	81.74 ± 1.92	72.84 ± 1.89
SG-UNet [21]	85.81 ± 1.05	82.32 ± 1.05	74.82 ± 1.53
DPEGNet [22]	85.98 ± 0.82	82.44 ± 0.83	75.96 ± 0.79
Asymmetric-HybridModel [20]	91.95 ± 0.81	83.75 ± 0.82	76.74 ± 0.77
The proposed method	97.96 ± 0.72 ↑	94.30 ± 0.65 ↑	81.33 ± 0.61 ↑

**Table 4:** NSD results (in %) using images from the KiTS-2023 database.

Method	NSD for Kidneys	NSD for Tumors	NSD for Cysts
PSTUDA [16]	82.03 ± 1.75	72.04 ± 1.14	63.58 ± 1.25
DeepLabV3 and Xception65 [15]	83.12 ± 1.64	72.40 ± 1.97	64.18 ± 1.84
RenalSegNet [17]	83.43 ± 1.62	76.48 ± 1.94	66.11 ± 1.83
Dual-stage segmentation [18]	83.67 ± 1.48	78.31 ± 1.90	67.62 ± 1.80
Swin-IBNet [19]	84.01 ± 1.15	79.55 ± 1.88	70.64 ± 1.77
SG-UNet [21]	84.68 ± 1.01	81.25 ± 1.03	72.24 ± 1.24
DPEGNet [22]	84.93 ± 0.75	81.59 ± 0.77	73.11 ± 0.68
Asymmetric-HybridModel [20]	90.19 ± 0.75	82.89 ± 0.71	74.98 ± 0.63
The proposed method	97.22 ± 0.58 ↑	93.56 ± 0.52 ↑	80.59 ± 0.57 ↑

**Table 5:** DSC results (in %) using images from the Kaggle website.

Method	DSC for Kidneys	DSC for Tumors	DSC for Cysts
PSTUDA [16]	84.98 ± 1.76	79.49 ± 1.68	66.82 ± 1.77
DeepLabV3 and Xception65 [15]	85.03 ± 1.99	80.96 ± 1.98	68.89 ± 1.95
RenalSegNet [17]	85.12 ± 1.86	81.01 ± 1.95	69.24 ± 1.89
Dual-stage segmentation [18]	85.97 ± 1.83	81.98 ± 1.91	71.45 ± 1.84
Swin-IBNet [19]	86.01 ± 1.47	82.96 ± 1.91	73.04 ± 1.82
SG-UNet [21]	86.16 ± 0.85	83.47 ± 1.35	75.36 ± 1.79
DPEGNet [22]	86.82 ± 0.80	83.97 ± 0.72	76.14 ± 0.31
Asymmetric-HybridModel [20]	92.08 ± 0.66	84.14 ± 0.51	78.21 ± 0.31
The proposed method	98.74 ± 0.58 ↑	95.46 ± 0.48 ↑	82.64 ± 0.28 ↑

**Table 6:** NSD results (in %) using images from the Kaggle website.

Method	NSD for Kidneys	NSD for Tumors	NSD for Cysts
PSTUDA [16]	83.91 ± 1.24	75.43 ± 1.13	64.52 ± 1.75
DeepLabV3 and Xception65 [15]	84.01 ± 1.97	78.86 ± 1.95	65.97 ± 1.92
RenalSegNet [17]	84.25 ± 1.85	80.16 ± 1.91	66.01 ± 1.88
Dual-stage segmentation [18]	84.91 ± 1.83	80.96 ± 1.89	70.10 ± 1.83
Swin-IBNet [19]	85.03 ± 1.46	81.96 ± 1.81	71.02 ± 1.81
SG-UNet [21]	85.14 ± 0.85	82.11 ± 1.32	73.09 ± 1.77
DPEGNet [22]	85.68 ± 0.78	82.24 ± 0.72	75.82 ± 0.30
Asymmetric-HybridModel [20]	91.41 ± 0.65	83.62 ± 0.50	76.42 ± 0.29
The proposed method	98.02 ± 0.52 ↑	94.11 ± 0.47 ↑	81.26 ± 0.2 ↑

**Table 7:** DSC results (in %) using images from the KiTS-2023 and Kaggle databases.

Method	DSC for Kidneys	DSC for Tumors	DSC for Cysts
PSTUDA [16]	84.48 ± 1.62	79.02 ± 1.63	66.27 ± 1.71
DeepLabV3 and Xception65 [15]	84.91 ± 1.97	79.88 ± 1.99	67.74 ± 1.96
RenalSegNet [17]	84.97 ± 1.80	80.69 ± 1.96	69.12 ± 1.92
Dual-stage segmentation [18]	85.45 ± 1.67	81.43 ± 1.92	70.81 ± 1.88
Swin-IBNet [19]	85.87 ± 1.33	82.35 ± 1.91	72.94 ± 1.86
SG-UNet [21]	85.99 ± 0.95	82.90 ± 1.20	75.09 ± 1.66
DPEGNet [22]	86.40 ± 0.81	83.21 ± 0.76	76.05 ± 0.55
Asymmetric-HybridModel [20]	92.02 ± 0.73	83.95 ± 1.67	77.48 ± 0.54
The proposed method	98.35 ± 0.65 ↑	94.88 ± 0.57 ↑	81.99 ± 0.45 ↑

**Table 8:** NSD results (in %) using images from the KiTS-2023 and Kaggle databases.

Method	NSD for Kidneys	NSD for Tumors	NSD for Cysts
PSTUDA [16]	82.97 ± 1.50	73.76 ± 1.14	64.05 ± 1.5
DeepLabV3 and Xception65 [15]	83.57 ± 1.81	75.63 ± 1.96	65.08 ± 1.88
RenalSegNet [17]	83.84 ± 1.74	78.32 ± 1.93	66.06 ± 1.86
Dual-stage segmentation [18]	84.39 ± 1.66	79.64 ± 1.90	68.86 ± 1.82
Swin-IBNet [19]	84.52 ± 1.31	80.76 ± 1.84	70.83 ± 1.79
SG-UNet [21]	84.91 ± 0.93	81.68 ± 1.18	72.67 ± 1.51
DPEGNet [22]	85.31 ± 1.77	81.92 ± 0.75	74.47 ± 1.49
Asymmetric-HybridModel [20]	90.80 ± 0.70	83.26 ± 0.61	75.70 ± 1.46
The proposed method	97.62 ± 0.55 ↑	93.84 ± 0.50 ↑	80.93 ± 0.40 ↑

**Table 9:** Effectiveness of the DCA, CFF, ED, and PJST blocks using images from KiTS-2023.

DCA	CFF	ED	PJST	DSC for Kidneys (%)	DSC for Tumors (%)	DSC for Cysts (%)
─	─	─	─	71.26 ± 1.49	64.03 ± 1.54	56.08 ± 1.67
√	─	─	─	94.20 ± 1.37	90.74 ± 1.29	78.03 ± 1.48
√	√	─	─	95.77 ± 1.12	92.23 ± 1.18	79.49 ± 1.22
√	√	√	─	97.16 ± 0.87	93.60 ± 0.98	80.83 ± 0.79
√	√	√	√	98.35 ± 0.65	94.88 ± 0.57	81.99 ± 0.45

**Table 10:** Effectiveness of the methods in terms of training times.

Method	Training Time (Min)
PSTUDA [16]	73.3
DeepLabV3 and Xception65 [15]	50.0
RenalSegNet [17]	43.3
Dual-stage segmentation [18]	60.0
Swin-IBNet [19]	56.7
SG-UNet [21]	63.3
DPEGNet [22]	70.0
Asymmetric-HybridModel [20]	66.7
The proposed method	53.3

**Table 11:** Effectiveness of different loss functions.

Loss Function	DSC for Kidneys (%)	DSC for Tumors (%)	DSC for Cysts (%)
Cross-entropy	87.27 ± 0.59	84.09 ± 0.56	79.15 ± 0.27
Focal	86.45 ± 0.48	83.34 ± 0.84	77.43 ± 0.58
Dice	84.63 ± 0.36	82.27 ± 0.76	76.78 ± 0.43
Hybrid (Cross-entropy and Dice)	98.35 ± 0.65	94.88 ± 0.57	81.99 ± 0.45

**Table 12:** Differences between DCA and CFF modules from prior attention structures (CBAM/SE-Net).

Feature	SE-Net/CBAM	Proposed DCA	Proposed CFF
Attention scope	Global channel or spatial	Joint channel re-weighting with local texture sensitivity	Cross-resolution feature preservation
Input design	Single-scale features	Dual-branch channel gating for organ/stone signal separation	Feature set alignment for stone-level structure retention
Target motivation	General vision tasks or classification	Small-lesion enhancement under high noise	Multi-scale contextual fusion for segmentation

## Data Availability

The original data used in the study are openly available in [27,28].

## Abbreviations

The following abbreviations are used in this manuscript:

DSC	Dice Similarity Coefficient
NSD	Normalized Surface Distance
CT	Computed Tomography
CNN	Convolutional Neural Network
ReLU	Rectified Linear Unit
IoU	Intersection over Union
DCA	Dynamic Channel Attention
CFF	Complementary Fusion of the Features
ED	Edge Detection
PJST	Patch-Joining and Swin Transformer
L-ReLU	Leaky ReLU
SAM	Self-Attention Mechanism
MSAM	Multi-head SAM
W-MSAM	Window- MSAM
SW-MSAM	Shifted Window-MSAM
TP	True Positives
TN	True Negatives
FP	False Positives
FN	False Negatives
S	Segmentation Result
G	Ground Truth
